# The interindividual variability of sleep timing and circadian phase in humans is influenced by daytime and evening light conditions

**DOI:** 10.1038/s41598-021-92863-z

**Published:** 2021-07-01

**Authors:** C. Papatsimpa, L. J. M. Schlangen, K. C. H. J. Smolders, J.-P. M. G. Linnartz, Y. A. W. de Kort

**Affiliations:** 1 Department of Electrical Engineering, Eindhoven University of Technology, Eindhoven, The Netherlands; 2 Department of Industrial Engineering and Innovation Sciences, Eindhoven University of Technology, Eindhoven, The Netherlands; 3 Signify, Eindhoven, The Netherlands

**Keywords:** Computational models, Circadian rhythms and sleep

## Abstract

Human cognitive functioning shows circadian variations throughout the day. However, individuals largely differ in their timing during the day of when they are more capable of performing specific tasks and when they prefer to sleep. These interindividual differences in preferred temporal organization of sleep and daytime activities define the chronotype. Since a late chronotype is associated with adverse mental and physical consequences, it is of vital importance to study how lighting environments affect chronotype. Here, we use a mathematical model of the human circadian pacemaker to understand how light in the built environment changes the chronotype distribution in the population. In line with experimental findings, we show that when individuals spend their days in relatively dim light conditions, this not only results in a later phase of their biological clock but also increases interindividual differences in circadian phase angle of entrainment and preferred sleep timing. Increasing daytime illuminance results in a more narrow distribution of sleep timing and circadian phase, and this effect is more pronounced for longer photoperiods. The model results demonstrate that modern lifestyle changes the chronotype distribution towards more eveningness and more extreme differences in eveningness. Such model-based predictions can be used to design guidelines for workplace lighting that help limiting circadian phase differences, and craft new lighting strategies that support human performance, health and wellbeing.

## Introduction

The central biological clock in the brain has a near-24 h rhythmicity that is a main determinant of individuals’ daily rhythm of rest and activity. It also orchestrates the daily rhythms in human physiology and behavior such as the sleep/wake cycle, hormone secretion, and subjective alertness and performance^1^. As such, many aspects of human performance are reported to cycle with a 24 h rhythmicity. For example, sports and muscular performance are optimal in the subjective early evening^2^ and circadian rhythms have been found in neuropsychological processes such as attention, working memory, and executive functions^3^.

Individuals vary greatly in terms of their preferences for the timing of performing specific tasks and when they prefer to sleep and wake. These naturally occurring interindividual differences in preferred habitual sleep–wake timing are known as chronotype^4^. Chronotype is seen as the behavioral manifestation of an underlying circadian rhythm in real-life conditions. Early chronotypes naturally wake up early and prefer to go to sleep early, while late chronotypes generally display a relatively late timing of their sleep and wake episodes during the 24 h light–dark cycle. The phase difference established between a marker of an individual’s circadian rhythm (such as sleep timing, dim light melatonin onset, and core body temperature [CBT] minimum) and the entraining zeitgeber cycle is known as entrainment phase (*ψ*). Assessing phase relationships is an important tool for understanding the circadian machinery. Three factors are most likely to contribute to the interindividual differences in chronotype. The first one is genetics. In particular, genetic variation in the PER1 and PER3 period genes is associated with chronotype^5,6^, while the PER2 gene is associated with the intrinsic circadian period in humans and this variation in intrinsic circadian periods influences morning and evening preferences^7^; people with a longer circadian period tend to be later chronotypes while people with a shorter period tend to be earlier chronotypes. The other two chronotype-determining factors are a differing “zeitgeber” signal (in particular light exposure), and age (chronotype changes across the lifespan^8^). When extreme chronotypes are forced to adapt their activities to the social time, this may cause difficulties in participating in work, school, and other social activities. This mismatch between daily schedules and endogenous circadian rhythmicity can have a profound effect on people’s mental and physical health. In fact, there are more than 100 studies that relate circadian disruption to a wide variety of health risks and diseases, including mood disorders, depression, diabetes, obesity, cardiovascular disease, and cancer^9^.

For millennia, the human circadian system has evolved to secure entrainment to the natural 24 h pattern of day and night, with the temporal organization of natural light and darkness as the main zeitgeber^10,11^. However, this entrainment is disrupted by modern lifestyle. Currently, people spend on average 90% of their time indoors^12^ with typical indoor light levels during daytime that are much lower than the natural light outdoors. Daytime illuminance outdoors normally ranges between 2000 and 100,000 lx, whereas indoor lighting is usually significantly lower. For instance, the European standard for lighting of work places (CEN 2011^13^) specifies minimum values for maintained horizontal illuminance in offices between 200 and 750 lx, depending on the specific task. In practice, typical horizontal (desk) illuminance levels in European offices are reported to range between 75 and 2500 lx^14^. Vertical illuminances at the cornea are likely to be substantially lower, typically between 0.3 and 0.5 times the horizontal illuminance measured at desk level, depending on the directional properties of the light source(s). In fact, Smolders et al.^15^ report an average illuminance of 200 lx at the eye position during daytime hours. Additionally, the advent of electric light has allowed people to self-regulate their individual light exposures which often leads to extended light exposure after sunset. This unnatural light exposure compromises the stability and entrainment of circadian rhythms, with serious implications for sleep, health and performance. It has been shown that insufficient light exposure during the day and too much evening light exposure delays the circadian system and can acutely suppress melatonin levels and subjective sleepiness in the evening and at night^16–19^. Recent evidence has also revealed that there is more than a 50-fold difference in sensitivity to evening light for melatonin suppression across individuals^20^. Such a variability in sensitivity to light could also play a role in other responses to light. However, interindividual differences in the response of the circadian system to light exposure can become less distinct when individuals are exposed to stronger zeitgeber strength. For example, Wright et al.^21^, showed that a week of camping outdoors in summer under only natural light conditions advanced circadian phase and reduced circadian phase variability in a group of eight individuals. Their habitual luminous exposure patterns (i.e., when living in conditions with electric light) could be characterized as that of a weak and temporally confusing zeitgeber. Thus, understanding the mechanisms of human light entrainment is increasingly important for the development of lighting control systems that may reduce the numerous pathophysiological repercussions induced by chrono-disruption^22,23^.

A substantial body of research has used mathematical modeling to predict and understand chronotype in humans. Phillips et al.^24^ used a mathematical model that incorporates the effects of light, circadian rhythmicity and sleep homeostasis to examine how chronotype is affected by interindividual differences in physiological parameters, while Skeldon et al.^25^ used a modeling approach to quantify age-related changes in sleep timing and duration across lifespan. The theoretical impact of self-selection of light exposure on the entrainment of the human circadian system and sleep timing has also been studied. Skeldon et al.^26^ provided a mathematical framework to examine and quantify how access to self-selected light and social constrains delay circadian rhythmicity and sleep timing. Swaminathan et al.^27^ used mathematical modeling to show how intrinsic differences in sleep and circadian timing can be amplified by self-selected use of artificial light sources. They showed that access to artificial light may evoke more than double the variation in sleep timing compared to living under natural light conditions. Granada et al.^28^ used analytical models to study how the phase of entrainment depends on clock and Zeitgeber properties. They show that strong oscillators with a narrow entrainment range exhibit more flexible entrainment phases while large Zeitgeber signals lead to large entrainment range. Schmal et al.^29^ used a similar model to investigate how local conditions of natural light determine the range of entrainment across seasons and latitudes.

The goal of this work is to systematically study how light conditions and interindividual variations in intrinsic circadian period affect the circadian phase distribution within the general population. Previous work has mostly concentrated on modelling circadian phase on an individual basis and only explored circadian phase distributions on a population level for a few light conditions. We now present results of a systematic study covering a wide range of realistic light profiles, both under natural and self-selected conditions. In particular, we will study the effects of different 24 h light–dark cycles on the circadian phase distribution within the general population: (A) LD 16:8 (i.e., a 16 h photoperiod, L, and an 8 h scotoperiod, with photoperiod illuminances varying between 0 and 10,000 lx, (B) LD 10:14 schemes with photoperiod illuminances between 0 and 10,000 lx, (C) schemes with constant daytime illuminances (wake to 19:00) varying between 0 and 10,000 lx, in combination with 15 lx evening light exposure (19:00 to sleep), and (D) like in (C) but with daytime illuminances of 200, 800 and 2000 lx in combination with evening exposures ranging between 0 and 100 lx. The knowledge about the implications of light on circadian health is already evident^30–32^. With the use of analytically tractable models, we provide a mathematical framework to quantify these effects and understand how lighting in the built environment affects circadian timing and might amplify interindividual variations in chronotype, widening the difference in preferred timing of sleep and work schedules between early and late chronotypes. We strongly believe that such a quantified model is an essential ingredient to interpret and translate existing scientific insights towards intelligent, automated lighting control algorithms, as these need to serve a broader range of conditions and use cases than can realistically be tested in specific experimental studies.

## Results

### Dim photoperiods widen the distribution of entrainment phase in humans

**Figure 1:**
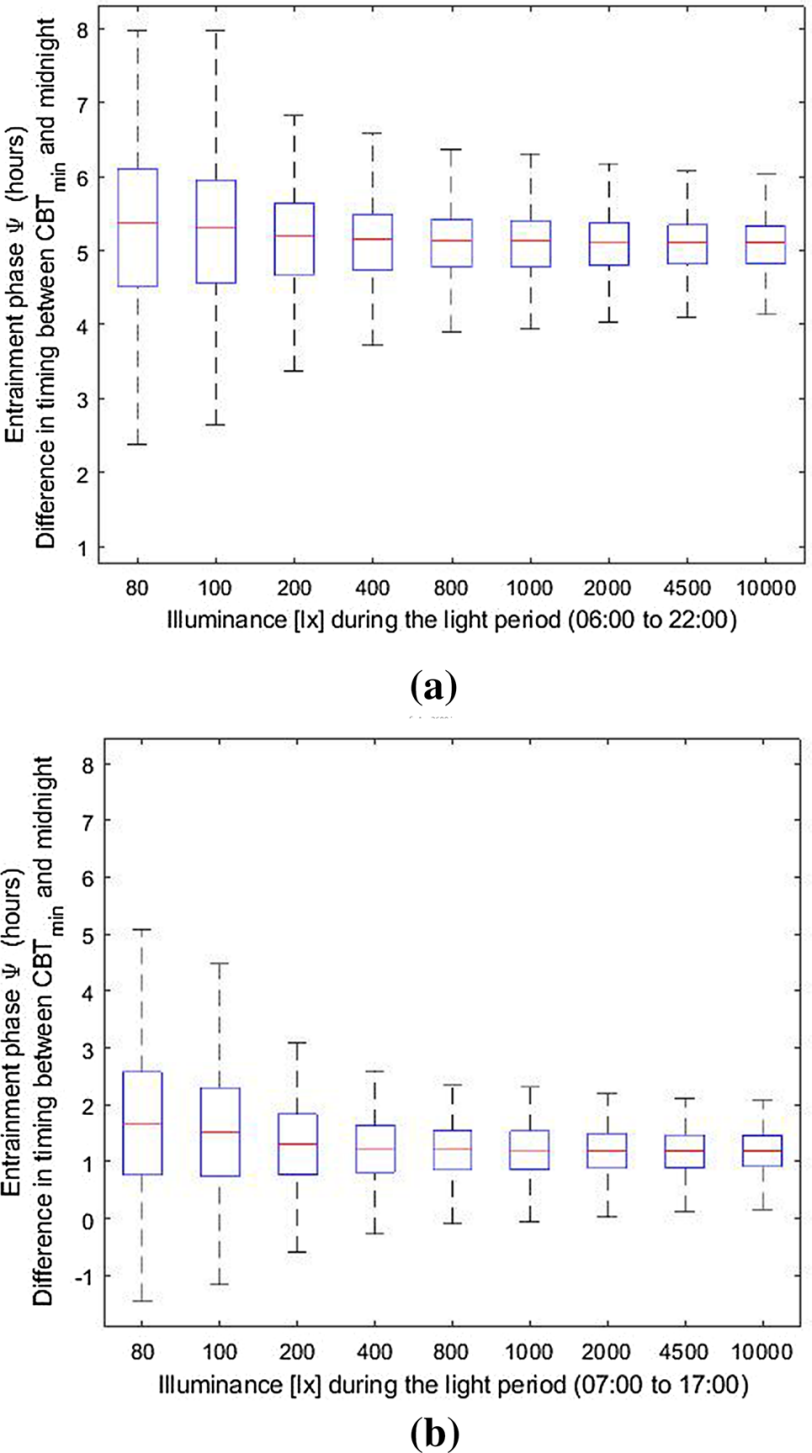
Entrainment phase angle distribution for a population of 200 simulated individuals with a normally distributed intrinsic circadian period with means (± SD) of 24.15 (± 0.2) h^33^ when entrained to various corneal illuminances (i.e., at the eye position). (**a**) Results for the LD 16:8 schedule. (**b**) Results for the LD 10:14 schedule. The central marker indicates the median, and the bottom and top edges indicate the 25th and 75th percentiles, respectively. The whiskers indicate the most extreme data points (population minima and maxima).

First, we considered a light–dark cycle with a 16 h photoperiod (06:00–22:00) at a constant illuminance and a scotoperiod of 8 h (denoted as LD 16:8) and calculated the distribution of the phase angle of circadian entrainment for different illuminances (Fig. 1a). Note that in order to investigate the effect of photoperiod length on the distribution of the phase angle of entrainment, we initially consider a fixed-length photoperiod where individuals can only influence the light pattern by sleeping, i.e., the available light is blocked during sleep episodes*.* We defined the phase angle of entrainment as the phase difference (in hours) between the timepoint of the nadir in body temperature (see “Methods”) and midnight. The model predicts that brighter photoperiods not only advance the circadian phase, but also reduce interindividual differences in circadian phase. At low illuminances of, for example, 100 lx at eye level, the entrainment phase shows a wide distribution with a mean (± SD) of 5.27 (± 1.36) h and range (difference between the largest and smallest value) of 6.23 h. This distribution is considerably narrowed as the light level increases. For example, at 800 lx the phase of entrainment has a mean (± SD) of 5.1 (± 0.3) and a range of 3.64 h. Under shorter photoperiods, the effect of higher light exposures on narrowing the phase distribution is less pronounced, but still considerable, see Fig. 1b. Here, we considered a light–dark cycle with a 10-h photoperiod (7:00–17:00) with a constant illuminance and a scotoperiod of 14 h (denoted as LD 10:14) and calculated the distribution of the phase of circadian entrainment for different illuminances. In the short photoperiod, exposure to 10,000 lx shows a distribution with mean (± SD) of 1.19 (± 0.15) h which is 74% less wide compared to exposure to 200 lx. In comparison, in the long photoperiod the distribution is narrowed down by 86% when comparing the 200 and 10,000 lx cases.

### Low daytime illuminances widen the differences between late and early chronotypes

**Figure 2:**
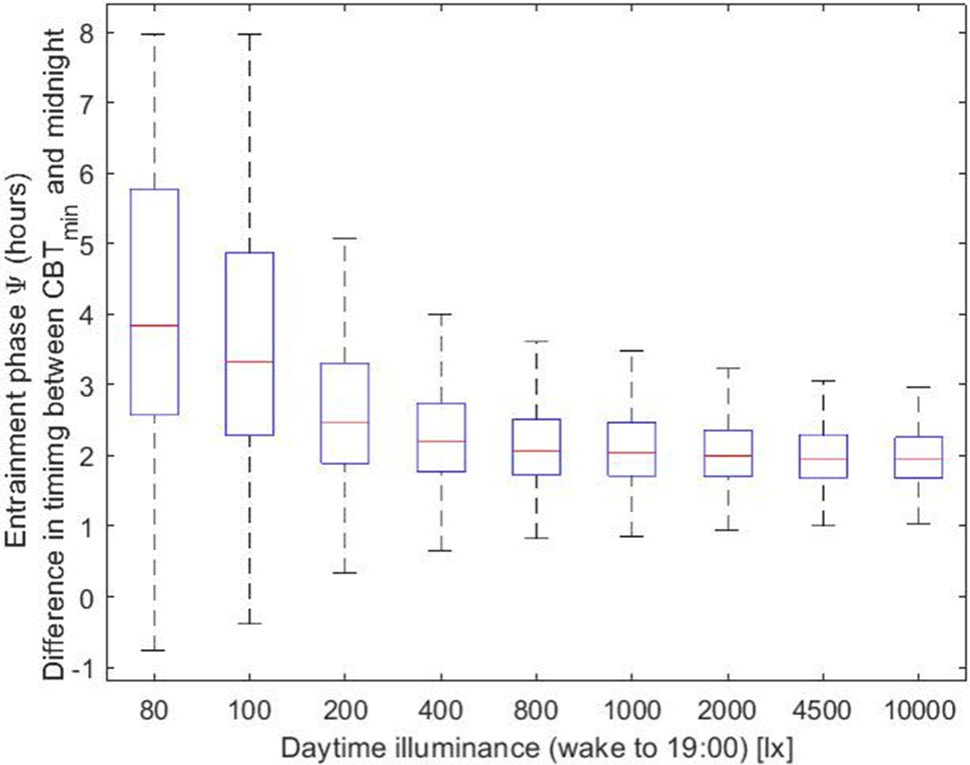
Entrainment phase angle distribution for a population of 200 simulated individuals with a normally distributed intrinsic circadian period with means (± SD) of 24.15 (± 0.2) h^33^. Illuminances refer to corneal light exposure (i.e., at the eye) for daytime (wake-19:00) illuminance (L_1_) set to the values indicated on the x-axis. Evening light exposure L_2_ was simulated at 30 lx from 19:00 until sleep onset. The central marker indicates the median, and the bottom and top edges indicate the 25th and 75th percentiles, respectively. The whiskers extend to the most extreme data points (population minima and maxima).

**Figure 3:**
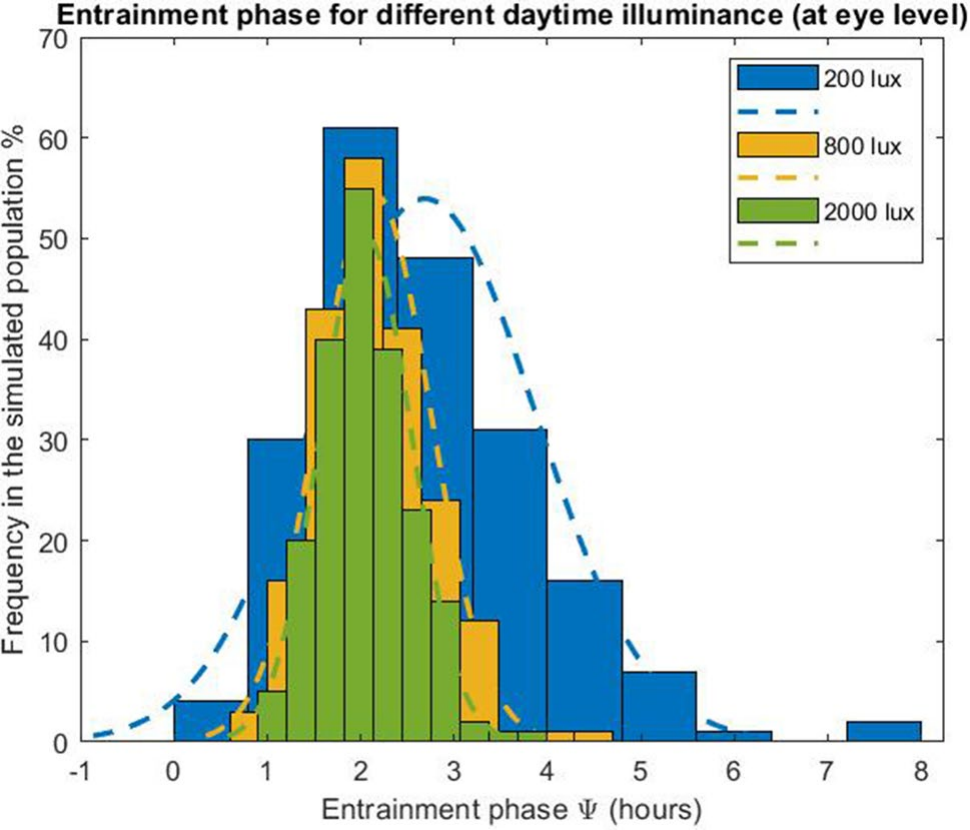
Entrainment phase angle distribution for a population of 200 simulated individuals with a normally distributed intrinsic circadian period with means (± SD) of 24.15 (± 0.2) h^33^. Illuminances represent the corneal light exposure (i.e., at the eye position). Results are presented for three different daytime (wake-19:00) illuminances (L_1_ = 200, 800 and 2000 lx at the eye), all with evening (19:00-sleep) illuminance L_2_ of 30 lx.

As a second case, we considered a light condition with evening light (L_2_) set at 30 lx and a constant daytime illuminance (L_1_) that was varied logarithmically between 0 and 10,000 lx. In order to mimic the self-selection of light in real life conditions, we consider that when the model wakes-up light levels are set to the daytime illuminance (up to 19:00) and to 30 lx from 19:00 up to the time that model falls asleep. We note that in the simulations the actual length of the photoperiod depends on the sleep–wake behavior; individuals with different intrinsic circadian period will hence generate different light profiles, due to different amount of time spent awake. Similar to the previous case, the model predicts that exposure to bright daytime light reduces interindividual differences in entrainment phase (Fig. 2), thus, bringing later chronotypes closer to earlier chronotypes. Even under typical office illuminances (~ 200 lx on average, measured vertically at the eye position), the phase of entrainment shows a rather wide distribution with a mean (± SD) of 2.68 (± 1.39) h. In fact, the phase angle range (i.e., the difference between the largest and smallest phase angle of entrainment) is 4.74 h (Fig. 3), meaning that extremely early and late chronotypes display a huge gap in the phase of their circadian rhythm. This interindividual variability is significantly reduced when humans are exposed to more natural, outdoor-like daytime light conditions, for instance when receiving 2000 lx at the eye. For 2000 lx, the mean phase of entrainment (± SD) is 2 (± 0.24) h, and the distribution shows a range of 2.28 h. These model results corroborate experimental studies that have shown that more natural daylight exposure reduces interindividual differences in circadian timing^21^.

### Evening light exposure delays circadian timing and increases eveningness

**Figure 4:**
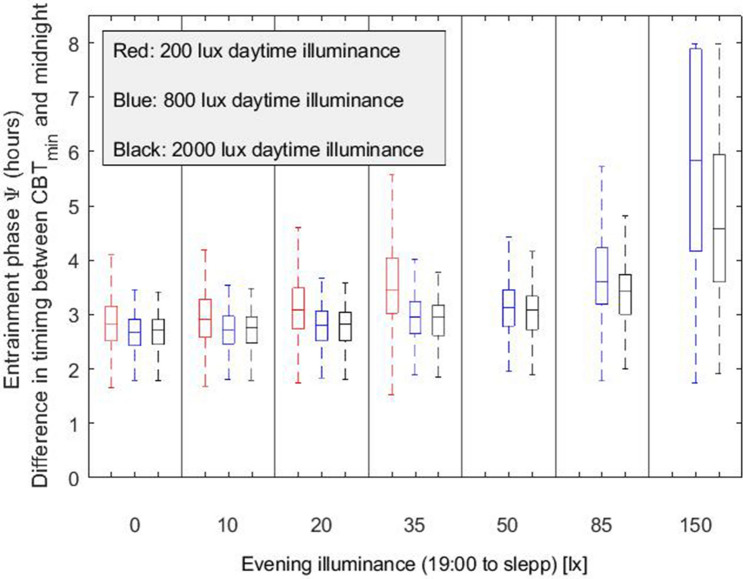
Entrainment phase angle distribution for a population of 200 simulated individuals with a normally distributed intrinsic circadian period with means (± SD) of 24.15 (± 0.2) h^33^. Illuminances represent the corneal light exposure (i.e., at the eye position). Results are presented for two different evening (19:00-sleep) illuminances of 10 and 35 lx, respectively, all with 200 lx daytime (wake-19:00) illuminance.

**Figure 5:**
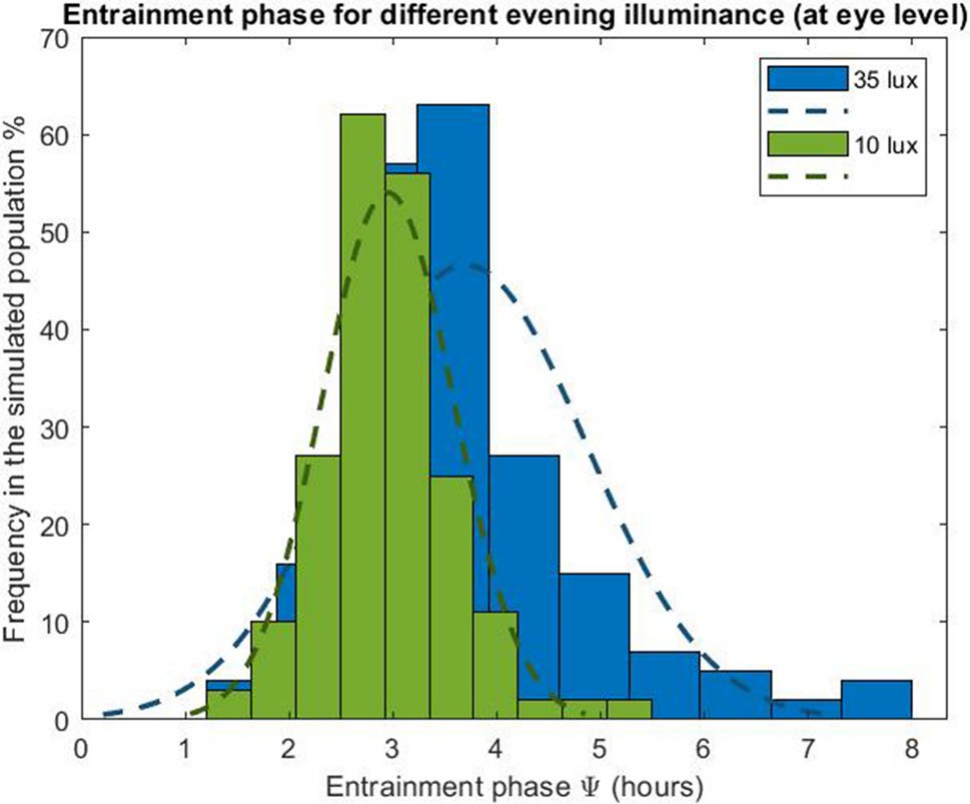
Entrainment phase angle distribution for a population of 200 simulated individuals with a normally distributed intrinsic circadian period with means (± SD) of 24.15 (± 0.2) h^33^. Illuminances refer to corneal light exposure (i.e., at the eye). Results are presented for daytime (wake-19:00) illuminances (L_1_) of 200, 800 and 2000 lx, and evening (19:00-sleep onset) illuminance (L_2_) set to the values indicated on the x-axis. The central marker indicates the median, and the bottom and top edges indicate the 25th and 75th percentiles, respectively. The whiskers extend to the most extreme data points (population minima and maxima). We note that in the absence of sufficient differentiation between daytime and evening light the model fails to entrain as also noted and discussed in^26^. Results are presented only for combinations of daytime-evening illuminances and intrinsic circadian periods for which the model entrains to 24-h rhythms.

**Figure 6:**
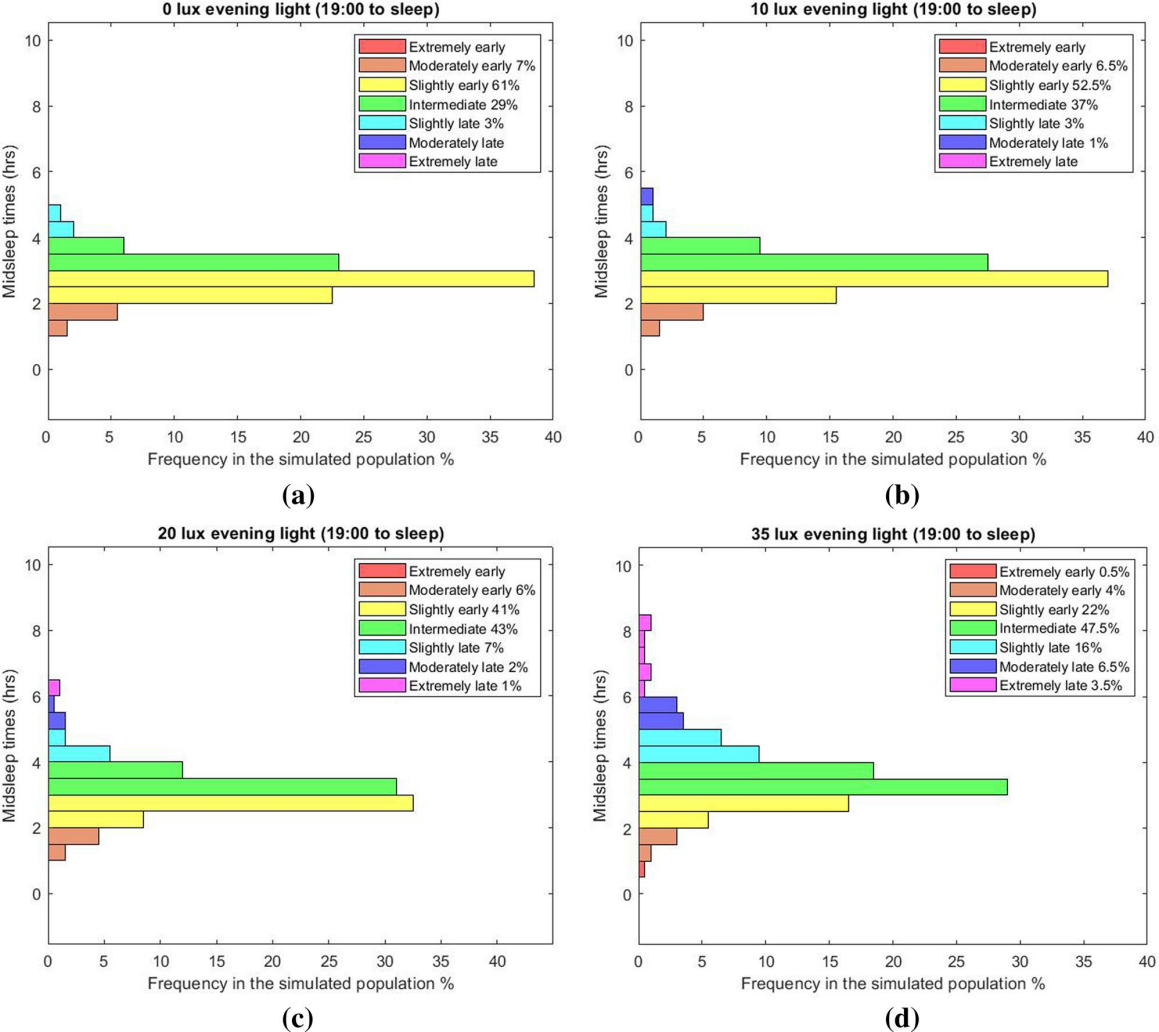
Model-predicted chronotype distribution under a constant daytime (wake-19:00) illuminance (L_1_) of 200 lx (at the eye) and various evening (19:00-sleep) illuminances (L_2_), all at the eye position: (**a**) 0 lx, (**b**) 10 lx, (**c**) 20 lx, and (**d**) 35 lx. The distributions are based on hourly bins. The population is classified into seven chronotypes indicated in the legends according to their midsleep timepoints.

In the third lighting scenario, we considered three different daytime (wake-19:00) illuminances (L_1_ = 200, 800 and 2000 lx respectively) and for each of them the evening (19:00-sleep) illuminance L_2_ was varied between 0 and 150 lx. The model predicts that for this range of evening illuminances, exposure to high levels of evening light increases the interindividual variation in the phase of entrainment. Under typical daytime illuminances (~ 200 lx) and low levels of evening illuminance, say 10 lx, the phase of entrainment shows a narrow distribution with mean (± SD) of 2.95 (± 0.4) h (Fig. 4). This interindividual variability is significantly increased when exposed to higher evening light levels, for instance when receiving 35 lx at the eye. For 35 lx, the mean phase of entrainment (± SD) is 3.76 (± 1.33) h. As expected, increasing the evening illuminance delays the timing of the core body temperature nadir for all daytime illuminances (Fig. 5). As a result, elevated evening illuminances push the whole population towards a later chronotype. Like in Fig. 2, higher daytime illuminances result in a more narrow and earlier chronotype distribution. Figure 6a–d show the chronotype distribution for 200 lx daytime (wake-19:00) illuminance and different evening (19:00-sleep) illuminances of 0, 20, 35 and 50 lx, respectively. The distributions classify the population according to their midsleep time value into seven groups as described by Roenneberg in^34^. In particular, midsleep times ≤ 01:00 $\text{c}$ orrespond to extremely early chronotypes, 01:00–02:00 to moderately early types, 02:00–03:00 to slightly early types, 03:00–04:00 to intermediate types, 04:00–05:00 to slightly late types, 05:00–06:00 to moderately late, and ≥ 06:00 to extremely late chronotypes. For relatively low evening illuminances (10 lx at the eye), the model predicts that the vast majority of the population has a slightly early and intermediate chronotype (52.5% and 37% of the simulated population, respectively). When exposed to brighter electrical light during the evening, the circadian phase of earlier chronotypes as well as late chronotypes become later, and as a result the whole population shifts towards more eveningness. For example, for evening illuminances of 35 lx (at the eye), a large part of the population shifts towards a late chronotype (i.e. 16% of the simulated population has a slightly late chronotype, 6.5% a moderately late chronotype and 3.5% an extremely late chronotype). For even higher evening light levels, e.g., 50 lx (at the eye), our simulations suggest that for the chosen intrinsic circadian period distribution, 45% of the population fails to remain entrained. Sufficient differentiation between daytime and evening light is required for the model to maintain entrainment^26^.

### Comparison to empirical data

We compared model predictions for the mean and standard deviation of sleep onset and offset timing to experimental data published in literature. For natural lighting conditions, we compare the experimental data reported in^21^ for summer natural 14 h 40 min:9 h 20 min light–dark cycle exposed to an average light exposure of 4487 ± 552 lx with a simulated 14.5 h photoperiod (06:00–20:30) with a constant illuminance set to 4500 lx and a scotoperiod of 9.5 h (LD 14.5:9.5). The standard deviation for model-predicted sleep onset and offset times are 0.2 h and 0.2 h, respectively, compared to experimental values of 0.3 h and 0.4 h. We note that the simulations consider an abrupt stepwise change to or from darkness, while in a realistic light profile light transitions (at dawn and dusk) occur more gradually. This might explain why the model predicts a narrower distribution (less early morning light leads to wider distributions).

For individuals living under realistic modern conditions (access to electric light), we compare the experimental data reported in^21^ with a simulated light profile (similar to the profile reported in^21^ for the electrical-lighting constructed environment) with morning light (wake-09:30) set to 100 lx, 5 h of bright light exposure at 1000 lx centered around midday (09:30–14:30) (^21^ reports 69% of waking day spent above 550 lx), afternoon (14:30–19:00) light set to 100 lx and evening light levels (19:00 to sleep) set to 20 lx (^21^ reports 30% of waking day spent below 50 lx and average light levels after sunset at 20 lx), The model predicts that sleep onset and offset values are pushed later compared to the natural light scenario, showing larger population variability. The standard deviations for the sleep onset and offset times were 1.06 h and 1.04 h for the model data and 1.4 h and 0.8 h for the experimental data. We note that model simulations have been performed by ranging the intrinsic circadian period according to a real distribution and assuming average values for the homeostatic parameters. However, Skeldon et al.^25^ have shown that changes in homeostatic model parameter µ, which represents the rate of homeostatic rise during wake, and ${v}_{vh}$, which represents the sensitivity to the homeostatic process, result in changes in model-predicted sleep timing and duration. This suggests that the model could fit the experimental data in^21^ more closely by tuning the homeostatic parameters.

Additionally, we compare the distribution of chronotypes reported in^4^ (MCTQ database based on almost 300,000 entries from all over the world) with the distributions reported by the model. We consider the simulated lighting schedule with 200 lx daytime (wake-19:00) and 35 lx evening (19:00-sleep) illuminance as the most representative of modern life conditions. The model predicts that 0.5% of the simulated population shows an extremely early chronotype, 4% moderately early, 22% slightly early, 47.5% intermediate, 16% slightly late, 6.5% moderately late and 3.5% shows an extremely late chronotype (Fig. 4d) compared to the corresponding experimental values of 1.2%, 5.9%, 22.3%, 29.8%, 21%, 11.7% and 8.2%, respectively. The model-predicted results show a more narrow distribution compared to experimental data. We note that the chronotype distribution predicted by the model is based on our assumption of what we would consider an average realistic light profile. We do not have data for the actual light profiles of participants in the study (which might greatly vary across season and geographic locations), neither is there data on the distribution of the intrinsic circadian period within^4^. Similarly to the previous case, the homeostatic model parameters were fixed to the average values reported in Table 1 and thus cannot represent the changes in sleep timing and duration across lifespan^25^. As a result, a one-to-one comparison with experimental data is not possible.

**Table 1:** Parameter values for the mathematical models.

Circadian model parameter	Value	Sleep model parameter	Value
$\mu$: parameter describing the stiffness (dampening) of the circadian oscillator (Eq. 1)	$0.13$	${\tau }_{v}$: decay time for the neuromodulator expressed by VLPO population (Eq. 6)	10 s
$q$: parameter that weights the effect of light (B) on phase and amplitude of the pacemaker (Eq. 2)	$1/3$	${v}_{vm}$: parameter weighting the input from VLPO population to MA population (Eq. 6)	2.1 mVs
$k$: parameter that represents the effect of light on the period of the pacemaker (Eq. 2)	$0.55$	${\tau }_{v}$: decay time for the neuromodulator expressed by MA population (Eq. 7)	10 s
$\tau$: parameter representing the intrinsic circadian period (Eq. 2)	24.2 h	${v}_{mv}$: parameter weighting the input from MA population to VLPO population (Eq. 7)	1.8 mVs
${a}_{0}$(Eq. 4)	$0.1$	${D}_{m}$: fixed wake-promoting drive (Eq. 7)	1.3 mV
$\beta$: parameter that describes the constant rate of photoreceptor deactivation (Eq. 5)	$0.007$	$\chi$: characteristic time for somnogen clearance (Eq. 9)	48 h
$G$: scaling constant (Eq. 5)	37	${\mu }_{H}$: parameter that describes the rate of homeostatic rise during wake (Eq. 9)	4 nMs
		${v}_{vc}$: parameter that represents the sensitivity of the sleep drive to the circadian process (Eq. 10)	2.9 mV
		${v}_{vh}$: parameter that represents the sensitivity of the sleep drive to the homeostatic process (Eq. 10)	1 mVnM^−1^
		${D}_{0}$: a constant background inhibitory input to the sleep promoting neurons (Eq. 10)	− 10.2 mV
		${Q}_{th}$: threshold value (Eq. 11)	1 s^−1^

## Discussion

People vary greatly in the timing of their physiological functions and their preference with respect to the timing of their intellectual and physical activities. Here, we use a mathematical model to study and quantify how genetic predisposition (i.e. variations in intrinsic circadian period) and light in the built environment interact to determine individual preferences for the timing of daily behavior, often referred to as chronotype.

People nowadays spend around 90% of their time indoors^12,35^, where the typical indoor environment is characterized by relatively modest light exposures during daytime, especially compared to outdoor natural light exposures. Our analysis reveals that exposure to more elevated daytime illuminances reduces interindividual differences in circadian timing, and effectively reduces chronotype differences within the population. A higher daytime illuminance results in a more narrow and earlier distribution of chronotype, it restricts the (delaying) effects of evening light exposure on circadian phase, physiology and sleep^36,37^, and is protective against extreme eveningness. An earlier chronotype is shown to be positively correlated with better physical and mental health, self-esteem and familial relationships^38^, and was a significant predictor of better school and attention test performance in adolescents^39–41^. This is in accordance with previous studies showing that more daytime light exposure is associated with increased sleep quality and mood^17,42^, while lower levels of light in the workplace are associated with compromised physiology^18^.

Modern lifestyle is also characterized by extended exposures to light in the late-evening hours and at night. Our results show that exposure to average levels of evening not only shifts the population towards later chronotypes, but also, increases interindividual differences between people. In particular, the model predicts that for typical evening illuminance at home of 35 lx (see Fig. 4d), more than 20% the population develops a late chronotype (16% slightly late, 6.5% moderately late and 3.5% extremely late) and shows a wide distribution with interindividual variation of 1.33 h. This reinforced evening preference may exacerbate circadian and sleep-related problems associated with later chronotypes. Research indicates that individuals with a late chronotype have less sleep quality and report higher levels of chronic work-related fatigue^31^, are at increased risk of experiencing emotional problems, including depressive symptoms^43,44^, while eveningness is also associated with metabolic disorders and body composition^45^ and increased likelihood of being a smoker, consuming alcohol, and caffeinated drinks^46^.

The model also quantitively replicates observed interindividual differences in chronotypes. Our simulation results are in line with experimental field studies that have shown that an increased (natural) light exposure during the summer reduces individual differences in circadian timing^21^. For shorter photoperiods, e.g. in wintertime, the effect of an increased (natural) light exposure on reducing interindividual differences in circadian phase is less pronounced, as shown experimentally in^47^. This is consistent with the current findings for the shorter day case, i.e., LD 10:14 schemes. This gives us confidence that mathematical models can be successfully used to test and develop chronobiological insights and translate these into recommendations and control strategies for real world lighting applications.

The oscillator model has been established in the late 90 s, a time period that the spectral sensitivity of the human eye was quantified in terms of red, green and blue cone sensitivity. However, current literature recognizes that photopic lux is limited in its ability to quantify the effects of light on the circadian system^48^. With the discovery of the intrinsically photosensitive retinal ganglion cells (ipRGCs) as the primary mediators of the non-image-forming effects of light in humans, it is now recognized that the circadian system is particularly sensitive to short-wavelength light^49^. Modifications of the model to incorporate the effects of the light spectrum on circadian phase resetting^50^ would likely provide more accurate estimations for the phase resetting, alerting and melatonin suppression responses to light^51^. However, at present there is little data available on the spectral variation in personal light exposure across the day. As an initial step one could replace the photopic illuminance in the model by the melanopic equivalent daylight illuminance (melanopic EDI). This is a standardized metric that quantifies light for its ability to activate the intrinsic (melanopsin-based) photoreception of ipRGCs^52^. A photopic illuminance can be easily converted into the corresponding melanopic EDI by multiplying the illuminance with the (average or expected) melanopic daylight efficacy ratio (melanopic DER). The melanopic DER represents an “M/P” ratio and equals the melanopic flux M of a light condition/exposure divided by its photopic luminous flux P^53^. It may be expected that using melanopic EDI as input to the model will only introduce changes in the absolute values of sleep and wake up times but will not significantly change the trends in the distributions, unless the light sources used diverge radically from daylight or from the fluorescent lights likely used in the empirical studies underlying the mathematical models. For electric light conditions a correlated color temperature of about 4000 K is quite common, and the melanopic DER for this type of lighting is about 0.6, while for natural light the melanopic DER is close to 1^52^. This implies that the contrast between electrical and natural light will be larger when using melanopic EDI as input to the model rather than photopic illuminance.

A further limitation of the current analyses relates to generalizability. The current analysis has concentrated on people with a regular day active schedule. For (rotating) shift workers, light exposure patterns are more variable and this warrants dedicated model simulations^54^.

Theory-informed predictions based on simulated populations can yield meaningful insights and guidelines for smart workplace lighting installations that help limiting circadian phase differences and extreme eveningness in a group of people that share the same workplace and working hours, and allow for lighting strategies that effectively mitigate circadian misalignment. For example, these model-based predictions may lead to renewed recommendations for health, supporting lighting regimes with brighter days and dimmer nights/evenings within the built environment. The results in Fig. 5 show that the median circadian phase is not too much affected by the daytime illuminance as long as the evening illuminance remains well below the daytime illuminance. However, it is worth noting that the circadian phase distribution progressively widens when the evening illuminance is higher and more similar to the daytime illuminance, see Fig. 5. Moreover, for domestic evening illuminances, an increase in daytime illuminance from 200 to 800 lx results in a more narrow circadian phase distribution. The findings in Fig. 5 show that having more daytime light exposure not only makes people less vulnerable to the sleep compromising, phase delaying action of increased evening light exposures, but it also helps restricting the enormous variability in sleep timing across the population. If one is interested in making the chronotype distribution within a group of people optimally homogeneous, this may require different strategies than counteracting the phase delays on an individual basis^55^. These finding are especially relevant in view of the contemporary focus on environmental sustainability that results in a drive to reduce indoor illuminance as to limit electricity consumption, while modern urban lifestyles make us spend less time outdoors so that we increasingly depend on our indoor environment for a healthy (i.e. strong and regular) light–dark cycle.

## Methods

### Model of the circadian pacemaker

To model the individual responses of the human circadian system to light exposure, we adopt a widely accepted model of the circadian pacemaker, namely the Jewett–Forger–Kronauer model^56^ with minor revisions for the light activation rate as proposed by^57^. The circadian process is modeled as a limit-cycle oscillator that, in the absence of external light stimuli, oscillates with an intrinsic period that is close but not exactly equal to 24 h. Through the presence of light stimuli the oscillator entrains to the 24 h light–dark cycle, establishing a stable phase relationship with it. It is well-known that timing signals other than light, such as food intake and exercise, can potentially contribute to circadian entrainment^58^. However, the influence of those non-photic cues is not the major focus of this work and would not likely significantly contribute to the effects being studied here. Thus, we chose to adopt the photic-version of the current model, initially neglecting the non-photic effects on circadian entrainment as captured in^57^. Due to interindividual differences in intrinsic circadian period, individuals also differ in their phase angle of entrainment under a particular light–dark cycle. If the circadian period is slightly shorter than 24 h—say, 23.8—then the period of the biological clock has to be delayed or lengthened by 12 min by means of daily time cues as to match the 24 h rotational cycle of the earth. If the intrinsic circadian period is longer than 24 h, it has to be advanced or shortened by daily time cues. Mathematically, the oscillation can be described by a pair of interacting state variables (*x*, *y*), defined by the continuous differential equations:

1 $$\dot{x}=\frac{\pi }{12}\left[y+\mu \left(\frac{1}{3}x+\frac{4}{3}{x}^{3}-\frac{256}{105}{x}^{7}\right)+B\right],$$

2 $$\dot{y}=\frac{\pi }{12}\left\{qBy-\left[{\left(\frac{24}{\tau 0.99729}\right)}^{2}+kB\right]x\right\},$$

where:

3 $$B=G\alpha \left(1-n\right)\left(1-bx\right)\left(1-by\right).$$

The intrinsic (endogenous) period of the oscillator is referred to as $\tau$, the stiffness (dampening) of the oscillator as $\mu$, and $n$ is the fraction of activated photoreceptors. The effect of light as a time cue is incorporated into the model through the light drive term $B$ to describe how the light intensity $I$ observed in the retina causes changes in the parameters of the circadian oscillator (speed and/or amplitude). A light and dark adaptation mechanism is incorporated in the model to describe the physiological process by which light initiates a chemical reaction within the photo-pigments of the retinal photoreceptors. This process can be thought of as comprising a pool of photoreceptors that can be either in the “used” state ($n$) or “ready” state (fraction $1-n$). The photoreceptors are activated by light at a rate $\alpha ,$ given by the updated model described in^57^, and deactivated with a constant rate $\beta$

4 $$\alpha ={a}_{0}\sqrt{\frac{I}{9500}}\frac{I}{I+100},$$

5 $$\dot{n}={\text{G}}\left[\alpha \left(1-n\right)-\beta n\right],$$

where the values of ${a}_{0}$, $\beta ,$ and $G$ have been determined from the experimental data.

The effect of light on the speed and amplitude of the circadian oscillator depends on the (internal) timing of light exposure. Light exposure during the late subjective night accelerates the pacemaker, resulting in a phase-advance, whereas light exposure during the early subjective night slows down the pacemaker, resulting in a phase-delay. To translate the model states into a physical indicator of the circadian state (a biomarker), a phase relationship between the model states and core body temperature was derived in^57^ such that

6 $$ {\text{time}}\;{\text{of}}\;CBT_{{min}}  = {\text{time}}\;{\text{of}}\;\varphi _{{yx}}  + 0.97\;{\text{h}} $$

where ${CBT}_{min}$ is the time at which the human core body temperature cycle reaches its nadir and ${\varphi }_{yx}$ is defined as the polar phase angle between state variables x and y such that.

7 $$\text{arctan}\frac{y}{x}=-170.7^\circ $$

### Entrainment phase

The entrainment phase angle ($\psi$) is the stable phase relationship between the internal pacemaker and the external day^59^. Here, we define the phase angle of entrainment as the time difference between a characteristic phase of the external day, namely midnight, and a characteristic phase of the internal circadian rhythm, namely at the timepoint of the nadir in core body temperature (CBT):

8 $$ \psi  = {\text{time}}\;{\text{of}}\;CBT_{{min}}  - {\text{midnight}}\;{\text{(00:00}}\;{\text{h)}}{\text{.}} $$

### Model of the sleep mechanism

In order to model the effects of light on the sleep–wake pattern, we adopt a modified version of the Phillips–Chen-Robinson model^24^ as proposed by Skeldon et al.^26^. In humans, sleep is regulated by two interacting, coupled mechanisms: the biological clock (the internal pacemaker) which generates a circadian rhythm in sleep–wake propensity, which we refer to as the circadian mechanism $C$, and a homeostatic process, $H,$ that represents the sleep dept which builds up during wakefulness and dissipates during sleep. Specifically, sleep and wake states occur as a result of mutual interaction between sleep promoting ($v$) and wake promoting ($m$) neurons that inhibit each other, as described by these equations for their mean electric potential, ${V}_{v}$ and ${V}_{m}$, respectively:

9 $${\tau }_{v}\dot{{V}_{v}}+{V}_{v}={-{v}_{vm}Q}_{m}+{D}_{v},$$

and

10 $${\tau }_{m}\dot{{V}_{m}}+{V}_{m}=-{v}_{mv}{Q}_{v}+{D}_{m}.$$

The relationship between the electric potential and firing rate of sleep and wake promoting neurons is described by

11 $${Q}_{j}=\frac{{Q}_{max}}{1+{\text{exp}}\left(\frac{\vartheta -{V}_{j}}{\sigma }\right)}$$

where neuron $j$ can be either of type $v$, for sleep-promoting, or type $m$, for wake-promoting. Here, ${Q}_{max}=100$ is the maximum possible firing rate, $\vartheta =10$ is the mean firing threshold, and $\sigma =3$ is the standard deviation of $\vartheta$. The parameters ${\tau }_{v,m}$ are the time constants of the neuronal process and the parameters ${v}_{vm,mv}$ weight the input from population $m$ to $v$ and $v$ to $m,$ respectively. The homeostatic process is directly correlated with the firing rate of the wake-promoting neurons and governed by the parameter ${\mu }_{H},$ which describes the rate of homeostatic rise during wakefulness.

12 $$ \chi \dot{H} + H = \mu _{H} Q_{m} . $$

The homeostatic dampening factor ${\mu }_{H}$ and circadian clock sensitivity ${v}_{vc}$ are age-related parameters that account for changes in sleep timing and duration across lifespan, but may be considered constant at a given age. The parameter $\chi$ is the time constant of the process. The drive of the sleep-promoting neurons, ${D}_{v}$, consists of both homeostatic and circadian components—namely:

13 $${D}_{v}={v}_{vc}C+{v}_{vh}H+{D}_{0},$$

where $H$ is the homeostatic sleep pressure, whose dynamics can be modelled as the level of a certain somnogenic agent such as adenosine, and $C$ represents the circadian factor. The parameter ${v}_{vh}$ is a constant which measures the sensitivity to the homeostatic process, ${v}_{vc}$ is the sensitivity to the circadian process, and ${D}_{0}$ is a constant offset. Finally, wake-up and sleep onset events are represented by the Heaviside step function, which means that a person wakes up if the firing rate of wake-promoting neurons, ${Q}_{m}$, is greater than a threshold value $Q_{{th}}  = 1\;{\text{s}}^{{ - 1}}$.

14 $$ S = {\mathcal{H}}\left( {Q_{m}  - Q_{{th}} } \right) = \left\{ {\begin{array}{*{20}l}    {1\left( {awake} \right),} \hfill & {{\text{if}}\;Q_{m}  \ge Q_{{th}} } \hfill  \\    {0\left( {sleeping} \right),} \hfill & {{\text{otherwise}}} \hfill  \\   \end{array} } \right.. $$

Consequently, spontaneous wake-up events are defined as the times (${t}_{w\_sp}$) that 0-to-1 transitions occur, while sleep onset events are defined as the times (${t}_{s\_sp}$) that 1-to-0 transitions occur.

15 $$ \begin{aligned}    & t_{{w\_sp}}  = t_{{S_{{0 - to - 1}} }}  \\     & t_{{s\_sp}}  = t_{{S_{{1 - to - 0}} }}  \\  \end{aligned} $$

In coupling the two models—i.e., the circadian rhythm model described by Eqs. (1)–(5) and the sleep/wake model described by Eqs. (6)–(12)—the circadian process $C$ is assumed to be approximately sinusoidal. The original model, developed by modelling the variations in the light and dark cycle as sinusoidal, assumes $C=\left(1+y\right)/2$. However, using realistic light profiles for the light intensity input, Skeldon et al.^26^ (suppl. mat) found that a phase shift was required to reproduce typically observed values for sleep duration and timing. We adopted a similar approach and used a phase-shifted version of $C$ to match real light intensity profiles and sleep–wake timings, as described in^60^—namely:

16 $$C=0.5\left(1+0.8x-0.55y\right).$$

All the model parameters are listed in Table 1.

### Entrainment simulations

**Figure 7:**
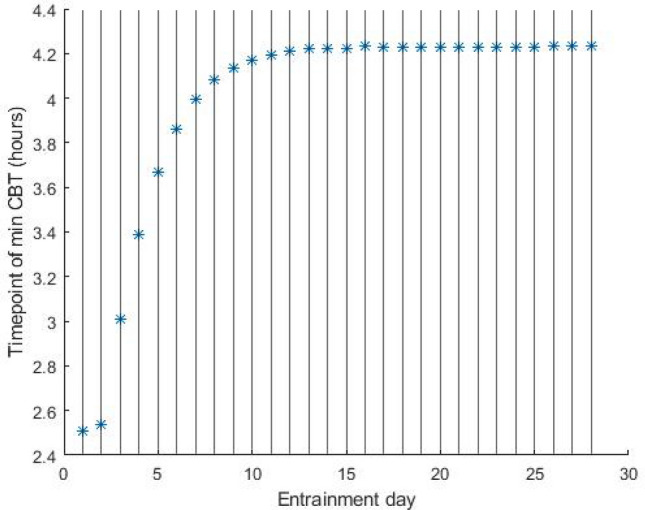
Example of daily shift in the predicted core body temperature minimum during entrainment. Results are presented for a 24 h light–dark cycle (LD 16:8 at 200 lx). Here, the model reaches a stable entrainment after 13 days. We consider that a stable entrainment has been reached if *CBTmind* − *CBTmind* − 1 < 0.01 ℎ. Initial state variable values (*x* and *y*) were set to − 0.9 and 0. 1, respectively, whereas variable *n* was set to 0.05.

Model simulations were performed in MATLAB (version R2020b). The Kronauer oscillator model entrains to a given light–dark cycle, thus establishing a stable phase (angle) relationship with the external zeitgeber. Typically, multiple weeks at a given light–dark cycle are required to establish a stable entrainment. Recovery from jet-lag involves a similar process. As described in^61^, 4 weeks of data provide sufficient time to guarantee stable entrainment. In our simulations, we used 4 weeks (28 days) of light data (30 min intervals) as input to the model. Model simulations are initiated on day 1 at the intended photoperiod/light pattern and run for 28 days. During the entrainment, the time of core-body temperature minimum gradually shifts towards an asymptote. In our simulations, the time of minimum core body temperature at the end of 28th day is considered stable and used to determine the entrainment phase angle that an individual establishes with the zeitgeber (time difference between min CBT and midnight). Figure 7 shows an example day-to-day shift in the predicted CBT minimum towards establishing a stable phase of entrainment. We note that in the absence of sufficient differentiation between daytime and evening light the model fails to entrain^26^. Results are presented only for combinations of daytime-evening illuminances and intrinsic circadian periods that the model entrains to 24-h rhythms.

### Light scenarios

Table 2 summarizes the different scenarios considered in our simulations. Firstly, we employed straightforward LD 16:8 and LD 10:14 schedules in which the illuminance is logarithmically varied from 0 to 10,000 lx. Individuals can only influence the light pattern by sleeping, i.e., the available light is blocked during sleep episodes. For the second set of simulations (Figs. 2 and 3), evening light (19:00 to sleep) is fixed to 30 lx and daytime light (wake to 19:00) is varied between 0 and 10.000 lx. The light profile is set to a fixed daytime level L_1_ upon wake-up and changes to an evening light level L_2_ at 19:00. Following an approach similar to^26^, the time at which lights are turned on/off is determined by when the model wakes up/goes to sleep and is thus ‘self-selected’. The choice of evening light level was motivated by typical levels in modern home lighting environments reported in^62^ (average melanopic EDI is 17.9 lx, SD = 13.6 lx) and assuming an M:P ratio of 0.55, while daytime light varied to cover the wide range of daytime illuminances for a range of contexts and environments. Subsequently, we fixed daytime light (wake to 19:00) while varying evening light (19:00 to sleep) between 0 and 150 lx. The choice of daytime light was motivated by the European standard for lighting of work places (CEN 2011^13^) which specifies minimum values for maintained horizontal illuminance in offices between 200 and 750 lx. Next to this we assumed a vertical to horizontal illuminance ratio of 0.5 for office fixtures, although in practice this ratio can strongly vary depending on variations in daylight entry and the inclination angle of the sun. The choice of evening light levels was motivated by^62^ that reported a melanopic EDI range from 3.9 to 77.4 lx (assuming an M:P ratio of 0.55). Finally, in order to compare model results with experimental data, we consider an LD 14.5:9.5 schedule in which illuminance is set to 4500 lx to simulate the natural light conditions reported in^21^. To simulate the case of electrical lighting in the constructed environment, we consider a scenario with morning light (wake-09:30) set to 100 lx, 5 h of bright light exposure at 1000 lx centered around midday (09:30–14:30), afternoon (14:30–19:00) light set to 100 lx and evening light levels (19:00 to sleep) set to 20 lx. Note that in all simulations, the mathematical model is based on corneal illuminances to characterize light exposure (i.e., the illuminance at the eye position measured on a plane of which the normal points towards the angle of gaze).

**Table 2:** Simulated light scenarios.

Scenario	Time interval	Illuminance (lx)
A. Long day, constant light (LD 16:8, Fig. 1a)	07:00–23:00	0 up to 10,000
	23:00–07:00	0
B. Short day, constant light (LD 10:14, Fig. 1b)	09:00–19:00	0 up to 10,000
	19:00–09:00	0
C. Long day with 20 lx evening light (Figs. 2 and 3)	Wake-19:00	0 up to 10,000
	19:00-sleep	15
D. Long day varying evening & daytime light exposure (Figs. 4, 5 and 6)	Wake-19:00	200, 800. 2000
	19:00-sleep	0 up to 150
E. Natural light scenario (LD 14.5:9.5)	06:00–20:30	4500
	20:20–06:00	0
F. Electrical-lighting in the constructed environment	Wake-09:30	100
	09;30–14:30	1000
	14:30–19:00	100
	19:00-sleep	20

### Distribution of intrinsic circadian period

It has been experimentally established that the intrinsic circadian period of the human circadian pacemaker shows interindividual variability with a period that is usually close to (but not exactly) 24 h, exhibiting, however a tight distribution consistent with other species (percent coefficients of variation (PCVs) of only 0.8%)^63^. Additionally, it has been demonstrated that the intrinsic circadian period is significantly shorter in women [24.09 ± 0.2 h (24 h 5 min ± 12 min)] than in men [24.19 ± 0.2 h (24 h 11 min ± 12 min); P < 0.01] and that a significantly greater proportion of women has an intrinsic circadian period shorter than 24.0 h (35% vs. 14%; P < 0.01)^33^. To investigate how variations in intrinsic circadian period may produce variations in the phase angle of entrainment between the internal clock and various external LD cycles, we varied model parameter $\tau$ representing the intrinsic period of the circadian oscillation model for an individual person. We simulated a population of 200 digital persons (100 men and 100 women). For each gender, the intrinsic period ($\tau$) of a digital person was drawn from a known as reported in literature for a sample of 105 males and 52 females^33^, while keeping all other model parameters fixed at their average values as listed in Table 1.

## Data Availability

No datasets were generated or analyzed during the current study.
